# Sensory-motor training versus resistance training among patients with knee osteoarthritis: randomized single-blind controlled trial

**DOI:** 10.1590/1516-3180.2017.0174100917

**Published:** 2017-12-07

**Authors:** Aline Bassoli Gomiero, Andrea Kayo, Marcelo Abraão, Maria Stella Peccin, Antonio Jose Grande, Virginia Fernandes Trevisani

**Affiliations:** I MSc. Physiotherapist, Department of Medicine, Universidade Federal de São Paulo (UNIFESP), São Paulo (SP), Brazil.; II PhD. Physiotherapist, Department of Medicine, Universidade Federal de São Paulo (UNIFESP), São Paulo (SP), Brazil.; III PhD. Associate Professor, Department of Medicine, Universidade Estadual de Mato Grosso do Sul (UEMS), Campo Grande (MS), Brazil.; IV MD, PhD. Associate Professor, Department of Medicine, Universidade Federal de São Paulo (UNIFESP), and Full Professor, Discipline of Reumatology, Universidade de Santo Amaro (UNISA), São Paulo (SP), Brazil.

**Keywords:** Osteoarthritis, Knee, Exercise, Pain, Randomized controlled trial

## Abstract

**BACKGROUND:**

Osteoarthritis of the knee is defined as a progressive disease of the synovial joints and is characterized by failure of joint damage repair. The objective here was to compare the effectiveness of sensory-motor training versus resistance training among patients with knee osteoarthritis.

**DESIGN AND SETTING:**

Randomized, single-blinded controlled trial conducted at the outpatient service of the University of Santo Amaro.

**METHODS:**

A total of 64 patients were randomly assigned to sensory-motor training or resistance training. The evaluations were performed at baseline and 16 weeks after the intervention and included pain evaluation on a visual analogue scale, isometric quadriceps femoris force measurement using a dynamometer, Timed Up and Go test, Tinetti balance scale, Western Ontario and McMaster Universities osteoarthritis index, and the SF-36 quality-of-life questionnaire. Data analysis was performed using analysis of variance with repeated measurements and Cohen’s effect size.

**RESULTS:**

Sensory-motor training may be a plausible alternative and showed a small effect on pain and a medium effect on maximal voluntary isometric contraction. Resistance training showed a small effect on balance and a medium effect on mobility.

**CONCLUSION:**

Resistance training and sensory motor training for the lower limbs among patients with knee osteoarthritis seemed to present similar effects on pain and function. However, because there was a considerable risk of type 2 error, further randomized clinical trials are still needed to provide a sound conclusion.

## INTRODUCTION

Osteoarthritis of the knee is defined as a progressive disease of the synovial joints and is characterized by failure of joint damage repair subsequent to stress that may have been initiated by an abnormality in any of the articular synovial tissues, including articular cartilage, subchondral bone, ligaments, menisci, periarticular muscles, peripheral nerves or synovia. This structural damage results in collapse of the cartilage and subchondral bone, thus leading to symptoms of pain, stiffness and dysfunction.^1^^,^^2^^,^^3^

The treatment options for knee osteoarthritis include non-pharmacological, pharmacological or surgical measures. Current clinical guidelines recommend non-pharmacological conservative strategies, including physical exercises, given their ease of application, small number of potential adverse effects and relatively low cost.^3^^,^^4^

Because of the large body of evidence demonstrating the beneficial effects of physical exercise among patients with osteoarthritis, exercise is often indicated as one of the main components in the rehabilitation process.^1^^,^^3^^,^^5^^,^^6^^,^^7^

Among the several types of physical exercise programs, muscle strengthening is important because of the relationship between muscle weakness, pain and poor function.^6^^,^^8^^,^^9^^,^^10^^,^^11^ However, traditional strengthening exercises may be insufficient for the subgroup of patients with functional joint instability. One study on this subgroup of patients investigated interventions focusing specifically on symptoms, thus maximizing the effectiveness of the rehabilitation program.^12^^,^^13^^,^^14^

As osteoarthritis progresses, sensory-motor skills such as proprioception, static and dynamic balance and neuromuscular control decline because of diminished daily physical exercises and increasing perception of pain. Thus, programs that include agility, coordination and balance (sensory-motor training) may be effective through exposing these individuals to potentially destabilizing loads. This allows the neuromuscular system to adapt to conditions that could induce knee instability during activities of daily living.^3^^,^^10^^,^^12^^,^^15^

In this light, the objective of this study was to compare the effectiveness of sensory-motor training (SMT) versus resistance training (RT) for relieving pain and improving function among a group of individuals with knee osteoarthritis, who were evaluated at the baseline and after 16 weeks of intervention. A secondary objective was to evaluate these individuals’ isometric strength, balance and general health.

## METHODS

### Trial design

This was a randomized single-blind controlled trial that was registered at ClinicalTrials (http://www.clinicaltrials.gov), under protocol number NCT01529398. All participants signed an informed consent form before they were included in the study. All procedures conducted in this study followed international standards for research on human beings, in accordance with the code of ethics of the World Medical Association (Declaration of Helsinki) for experiments involving humans.

### Participants

This study was conducted in the physiotherapy sector of the ambulatory of the Interlagos medical specialties outpatient service, which belongs to the University of Santo Amaro (Universidade de Santo Amaro, UNISA) in São Paulo, Brazil. It was conducted between March 2008 and July 2009.

The inclusion criteria were that the patients needed to have a diagnosis of tibiofemoral osteoarthritis that fulfilled the clinical criteria for knee osteoarthritis of the American College of Rheumatology (ACR),^16^ 1986; be between 50 and 75 years of age; have not done any physical activity for at least 3 months; and have reached a minimum educational level of 4^th^ grade of elementary education. Participants who presented the following were excluded: uncontrolled arterial hypertension; decompensated diabetes mellitus; decompensated thyroid diseases; cardiorespiratory diseases (ischemia, arrhythmia, precordial pain or physical exercise-induced bronchospasm); liver abnormalities; grade IV functional impairment (Kellgren-Lawrence radiographic scale); or other rheumatic diseases. In addition, patients who needed ambulatory devices and those who were on sick leave from work approved by the government agency for national insurance or presented any other related factor were also excluded.

### Interventions

Participants were allocated in a 1:1 ratio to either resistance training or sensory-motor training. Those assigned to the resistance training group received a 16-week exercise program twice a week, which included warm-up on a stationary bicycle for 10 minutes, quadriceps and hamstring strengthening exercises using ankle weights, isometric exercises for the quadriceps muscle (hip flexion with leg extended) and stretching for the lower limbs (stretching of the quadriceps, hamstrings and triceps surae). All physical exercises were performed bilaterally and at a volume of three sets of ten maximal repetitions.

The participants who were allocated to the SMT group received the same warm-up and stretching program as the RT group, with the same duration and frequency of treatment, but with replacement of the strengthening program with a program emphasizing agility, coordination and balance. This program included walking in different directions following verbal commands from the therapist; crossing steps while walking; crossing steps while walking backwards; implementing sudden changes of direction; walking on several types of surfaces (including mattresses); maintaining posture during use of a balance board; and using a mini-trampoline to expose individuals to potentially destabilizing loads.

To ensure linearity within the protocols, only one therapist supervised all the interventions. The groups were composed of four to five patients each, so that it was possible to supervise and monitor all the patients in a safe and effective manner.

In addition to the interventions described above, the two groups had concomitant intervention such as informative talks. They also received an educational program on knee osteoarthritis, which allowed the patients to clarify their doubts and concerns about the disease.

### Outcomes

#### Primary outcomes

Pain was one of the primary outcomes assessed. We used a visual analogue pain scale (VAS) for the participants to report the worst pain that they had felt in their knees over the last 24 hours prior to the evaluation. The VAS is an instrument based on a straight line (100 mm long) graduated from 0 to 100, on which the patient marks the intensity of his/her pain: from zero = no pain to 100 = worst pain imaginable. Table 1 presents the results from each outcome studied.

**Table 1:** Sensory-motor training versus resistance training among patients with knee osteoarthritis

Variables measured		Group				P* (Interaction effect)	P^**†**^ (Time [T0 versus T16])	Cohen’s d
		SMT ($\overset{-}{x}$)	95% CI	RT ($\overset{-}{x}$)	95% CI			Effect size post-intervention
VAS (score)	T0	6.3	5.47-7.13	6.7	5.80-7.60	0.702	< 0.001	0.24
	T16	4.6	3.84-5.36	4.1	3.16-5.04			
TUG (seconds)	T0	9.1	7.91-10.29	10.5	8.99-12.01	0.395	< 0.001	-0.67
	T16	7.9	7.47-8.33	8.7	7.69-9.71			
MVIC (kilograms)	T0	29.2	25.67-32.73	26.7	23.24-30.16	0.070	0.001	0.55
	T16	39.9	35.61-44.19	33.4	28.35-38.45			
Tinetti (score)	T0	24.3	22.64-25.96	24.1	21.97-26.23	0.832	0.001	- 0.22
	T16	26.0	25.17-26.83	26.5	25.74-27.26			
WOMAC (score)	T0	36.3	29.13-43.47	37.8	31.74-43.86	0.832	0.001	0.09
	T16	30.6	24.25-36.95	29.0	23.27-34.73			

SMT = sensory-motor training; CI = confidence interval; RT = resistance training; VAS = visual analogue scale; TUG = Timed Up and Go test; TINETTI = Tinetti balance assessment tool; MVIC = maximal voluntary isometric contraction; WOMAC = Western Ontario and McMaster Universities osteoarthritis index; *analysis of variance (ANOVA) for repeated measurements; ^†^t test conducted to compare means; T0 = baseline; T16 =16 weeks post-intervention.

Quality of life was assessed by means of the Short Form-36 quality-of-life questionnaire (SF-36). In this, zero points corresponds to the worst quality of life and 100 points corresponds to the best quality of life, as put forward through the questionnaire.^16^ The evaluations were performed at the baseline (T0) and after the end of the physical exercise program (T16).

The other primary outcome was mobility, as measured using the Timed Up and Go (TUG) test. In this test, we measured the time (in seconds) that it took for each individual to stand up from a chair, walk a distance of three meters in a straight line, turn around, walk back and sit down on the chair again.^17^ The outcome of mobility involves the individual’s gait speed, balance, functional level and ability to stand up, and the risk of falling to which this individual is exposed.

The evaluations were performed at the baseline (T0) and after the end of the physical exercise program (T16).

#### Secondary outcomes

The isometric strength of the quadriceps muscle (IS) was measured with the subject seated on an extensor chair, with isometric fixation at 45 degrees using a load cell for Miotec traction.

Balance and gait were evaluated through the Tinetti balance assessment tool. This test classifies gait parameters such as speed, step distance, symmetry, standing balance and spin, and also assesses changes to footing that are made while the subject’s eyes are closed. The maximum scores are 12 points for gait and 16 points for body balance, thus totaling 28 points.^18^

Functional capacity was measured by means of the Western Ontario and McMaster Universities (WOMAC) index questionnaire. This is specific for patients with knee osteoarthritis and provides information on pain, stiffness and physical function among these individuals. Higher scores indicate greater degree of severity of the disease.

### Sample size

The expected difference between the groups was 10%, considering the outcome of mobility, i.e. one point in the TUG test. Thus, a significance level of 5% and a test power of 80% were considered for the sample size calculation. This showed that the ideal sample size would be 46 patients for each group. However, we were only able to recruit 32 patients for each group, and thus the power of the study decreased to 68.3%.

### Randomization

The GraphPad Statmate 1.0 software was used to generate randomized numbers. The sequential numbers thus generated through the computerized randomization were then placed in opaque envelopes that were sealed by a physiotherapist who was not involved in the study, thereby keeping the allocation concealed. As the patients underwent their initial assessments, the same physiotherapist assigned the subject to one of the groups based on instructions from the next sealed envelope of the sequence.

### Blinding

A second physiotherapist sealed all the envelopes and participated in the evaluations on the study participants. Thus, she was blind to both treatment groups. Regarding the physiotherapist performing the interventions, she could not be blind because of the characteristics of the intervention. Likewise, the participants could not be blind due to the characteristics of the intervention.

### Statistical methods

All analyses were performed following the principles of intention-to-treat (ITT) analysis. In case of missing data, we used “last observation carried forward” as the imputation data method. The chi-square test was used to compare the groups regarding the qualitative variables (gender and Kellgren classification), while the descriptive variables were presented as absolute frequency (n) and relative frequency (%). The significance level was taken to be 5%.

For the quantitative variables of age, weight, height and body mass index (BMI), the paired Student t test was used, and the results were presented as summary measurements (i.e. the mean). In comparing quantitative variables between the groups, analysis of variance (ANOVA) for repeated measurements was used, and significant differences were taken to exist when P < 0.05. In situations in which there was an interaction effect, multiple comparisons were made to identify the differences found. We also estimated Cohen’s d effect size index. We classified the Cohen effect sizes as small (d = 0.2), medium (d = 0.5) or large (d ≥ 0.8).

There were no changes to the study protocol after the study started. Thus, the study registration protocol was followed exactly as it was written.

## RESULTS

### Participant flow

Over a six-month period, 120 patients with a diagnosis of knee osteoarthritis were attended at the rheumatology service of the Interlagos specialty clinic. Of these, 96 met the inclusion criteria for this study and were invited to participate in it.

**Figure 1:**
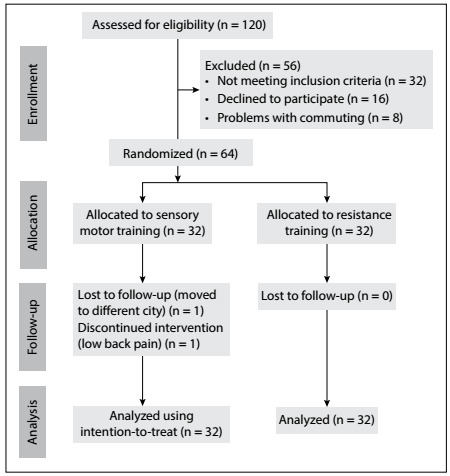
Flow diagram of the progress through the phases of the study.

Fifty-six did not meet the inclusion criteria (Figure 1). Thus, 64 participants were randomized to SMT or RT, to form two groups of 32 participants each. There was one loss from the follow-up in the sensory motor group because this participant moved to another city and one loss due to low back pain. In the resistance training group, there were no losses from the follow-up. The clinical and demographic characteristics of the participants are shown in Table 2.

**Table 2:** Participants’ characteristics

Variables	Groups				P*
	SMT		RT		
	$\overset{-}{x}$	SD	$\overset{-}{x}$	SD	
Age	61.6	6.8	61.8	6.4	0.895
Height	1.57	0.08	1.59	0.07	0.361
Weight	75.7	13	75.5	12.7	0.949
BMI	24.1	3.8	23.6	3.5	0.587
	n	%	n	%	
Gender					
Male	2	6.3%	1	3.1%	> 0.999
Female	30	93.8%	31	96.9%	
Hypertension^†^					
Yes	17	53.1%	20	62.5	0.448
No	15	46.9%	12	37.5	
Diabetes^†^ mellitus					
Yes	10	68.8%	4	12.5%	0.070
No	22	31.3%	28	87.5%	
Knee osteoarthritis					
Kellgren and Lawrence (K&L)					
Grade I	15	46.9%	13	40.6%	0.936
Grade II	9	28.1%	9	28.1%	
Grade III	6	18.8%	7	21.9%	
Grade IV	2	6.3%	3	9.4%	

SMT = sensory-motor training; RT = resistance training; SD = standard deviation; BMI = body mass index. *t test conducted to compare means; chi-square test used to compare proportions. ^†^self-reported by the participants, but diagnosed previously by a physician.

### Pain

The two groups were compared over time (T0 versus T16) and the interaction effect of baseline vs. post-intervention was measured. We observed that at T16, the RT group showed a greater reduction in the outcome of pain: mean difference, MD 0.50; 95% confidence interval, CI -0.66 to 1.66; and d = 0.24, i.e. a small effect.

### Quality of life

Regarding the SF-36 quality of life questionnaire, we found significant differences between the times evaluated, for the following subscales: physical role functioning, which was better in the RT group (MD 13.1; 95% CI 23.70 to 2.51; d = 0.14); vitality, which was better in the RT group (MD 13.9; 95% CI 24.00 to 3.80; d = 0.25, i.e. a small effect); emotional role functioning, which was better in the RT group (MD 36.5; 95% CI 55.53 to 17.47; d = -0.08); and mental health, which was better in the SMT group (MD 8.9; 95% CI 18.11 to 0.31; d = 0.52, i.e. a medium effect). In the SF-36 subscale of physical role functioning, there was an interaction effect between the groups, but there was only a statistically significant difference between the times evaluated for the RT group (P = 0.001). In the subscales of bodily pain, general health perception and social role functioning, we did not find any statistically significant difference between the groups or between the times evaluated. These results are shown in Table 3.

**Table 3:** Scores on the Short Form-36 (SF-36) questionnaire subscales

SF-36		Group				P* (Interaction effect)	P^**†**^ (Time [T0 versus T16])	Cohen’s d
		SMT ($\overset{-}{x}$)	95% CI	RT($\overset{-}{x}$)	95% CI			Effect size post-intervention
Physical role functioning	T0	51.4	43.22-59.58	38.3	31.70-44.90	0.034	0.001	0.14
	T16	54.8	46.26-63.34	51.4	42.57-60.23			
Physical functioning	T0	32.8	19.57-46.03	30.5	16.62-44.38	0.726	< 0.001	0.16
	T16	57.5	42.50-72.50	50.8	37.57-64.03			
Bodily pain	T0	50.4	40.41-60.39	48.0	38.81-57.19	0.886	0.060	0.18
	T16	59.3	50.14-68.46	54.8	45.75-63.85			
General health perceptions	T0	55.8	48.23-63.37	59.8	51.36-68.24	0.425	0.098	-0.06
	T16	60.8	53.88-67.72	62.0	54.57-69.43			
Vitality	T0	55.6	47.99-63.21	46.4	38.72-54.08	0.256	< 0.001	0.25
	T16	64.5	58.41-70.59	60.3	53.13-67.47			
Social role functioning	T0	72.8	62.63-82.97	70.8	59.80-81.80	0.465	0.932	0.29
	T16	74.0	65.78-82.22	67.3	57.89-76.71			
Emotional role functioning	T0	34.7	19.38-50.02	28.1	14.76-41.44	0.261	< 0.001	-0.08
	T16	61.1	46.25-75.96	64.6	49.96-79.24			
Mental health	T0	65.2	57.63-72.77	60.5	52.86-68.14	0.399	0.006	0.52
	T16	74.1	68.22-79.98	65.6	58.75-72.45			

SMT = sensory-motor training; CI = confidence interval; RT = resistance training. *Analysis of variance (ANOVA) for repeated measurements; ^†^paired t test conducted to compare means. T0 = baseline; T16 = 16 weeks post-intervention.

### Mobility

The two groups were compared at the baseline and after the intervention (T0 versus T16) and the interaction effect was measured. At T16, there were reductions in both groups regarding the outcome of mobility. The reduction was greater in the resistance training group: MD -0.80; 95% CI -1.85 to 0.25; d = -0.67, i.e. a medium effect.

### Isometric strength

The two groups were compared at the baseline and after the intervention (T0 versus T16) and the interaction effect was measured. At T16, the SMT group showed greater improvement in the outcome of isometric strength: MD 6.5; 95% CI 0.13 to 12.87; d = 0.55, i.e. a medium effect.

### Functional capacity

The two groups were compared at the baseline and after the intervention (T0 versus T16) and the interaction effect was measured. At T16, there were reductions in both groups regarding the outcome of functional capacity, although the reduction was greater in the resistance training group: MD -1.6; 95% CI -6.61 to 9.82; d = 0.09.

### Harm

One participant stopped the intervention and withdrew from the study due to low back pain.

## DISCUSSION

### Summary of main findings

Through searching the literature on this topic, and considering our personal experience, we saw that there was a need to investigate another type of physical exercise for treating osteoarthritis of the knee. In clinical practice, we had observed that not all patients benefited from resistance training, and that this type of exercise might be insufficient to achieve the desired improvements in quality of life and functionality. Thus, the purpose of this study was to compare the effectiveness of two separate types of physical exercise: sensory-motor training versus resistance training, in relation to improvement of pain and functioning within the study population.

In our study, we found significant differences between the groups, in the results relating to VAS, TUG, isometric strength, Tinetti balance scale, WOMAC questionnaire and the SF-36 subscales regarding physical aspects, vitality, emotional aspects and mental health. Thus, we observed improvements in pain, physical function and quality of life in both types of protocols proposed.

However, through estimating the Cohen’s d effect size, we observe that there were small effects towards the SMT group regarding pain measured using the VAS (d = 0.24) and regarding the SF-36 subscales of vitality (d = 0.25) and social role functioning (d = 0.29). There were medium effects regarding mental health (d = 0.52) and maximal voluntary isometric contraction (MVIC) (d = 0.55).

Regarding the effect size for resistance training, there was a medium effect on mobility (d = -0.67) and a small effect on balance (d = -0.22).

### Comparison with similar studies

We found only one other study^15^ that evaluated sensory-motor (proprioceptive) training among patients with knee osteoarthritis, and the findings from that study were similar to our results relating to pain. That study consisted of randomized controlled trial on 22 patients with bilateral knee osteoarthritis, in which a proprioceptive training program (n = 12) was compared with a control group (n = 10). The training group performed 11 different proprioceptive exercises for balance and coordination, twice a week for six weeks. This group that underwent sensory-motor training presented a significant reduction in perceived pain (as measured using a VAS) during activities of daily life and in performing functional tests after the physical exercise program (P < 0.05).

Using WOMAC, we observed that the effect size was insignificant. This agreed with the findings regarding pain and function from two studies^12^^,^^19^ in which sensory-motor training was performed in association with muscle-strengthening training, among patients with knee osteoarthritis. Diracoglu et al.^19^ compared the effectiveness of exercises for balance and kinesthesia training together with strengthening exercises versus strengthening exercises alone, among 60 participants. The patients received 24 training sessions over a period of eight weeks, and although the results indicated that both groups improved, there was no difference in the WOMAC questionnaire between the groups. The results from the SF-36 subscales of functional capacity, physical aspects and vitality and from the 10-minute walk test in the group that underwent balance and kinesthesia training were also better.^19^ Fitzgerald et al.^12^ compared a group of knee osteoarthritis patients who underwent a traditional training program (muscle strengthening of the lower limbs associated with stretching and joint amplitude gain exercises) with a group that performed the traditional program plus sensory training (agility and coordination). In both studies,^12^^,^^19^ although the groups that had additional sensory-motor training achieved improvements, the authors did not find any significant difference in comparison with the traditional rehabilitation program.

In our study, the isometric quadriceps strength improved in both groups. Although a medium effect size was observed in the SMT group, the values were not significantly different from those of the RT group after training. There were no changes in isokinetic strength in either group after the intervention period. We suggest that use of a proprioceptive exercise program may improve postural control and functional capacity, while decreasing perceived knee pain among patients with bilateral knee osteoarthritis.^12^^,^^19^^,^^20^

### Implication for further research and practice

Although it was hard to establish a comparison between our study and those cited above because of methodological differences, the results from our study suggest that sensory-motor training may be a plausible alternative, with a small effect towards this training in relation to pain and a medium effect in relation to MVIC. RT showed a small effect on balance and a medium effect on mobility. Regarding professional practice, we can hypothesize that the two types of exercises together can complement each other.

### Limitation of the study

One limitation of our study was the number of participants. In calculating the sample size, we reached a number that we were unable to recruit. Thus, the power of the study was reduced.

## CONCLUSION

Both resistance training for the lower limbs and sensory-motor training led to reduction of perceived pain and increased mobility in the study population. There were also improvements in functional capacity and isometric strength. The negative impact of knee osteoarthritis on the quality of life was attenuated through practicing any of the types of physical exercise training. 

Based on our findings, resistance training and sensory-motor training for the lower limbs among patients with knee osteoarthritis seemed to present similar effects on pain and function. However, because there was a considerable risk of type 2 error, further randomized clinical trials are still needed to provide a sound conclusion.
